# Spatial Navigation and Visuospatial Strategies in Typical and Atypical Aging

**DOI:** 10.3390/brainsci11111421

**Published:** 2021-10-27

**Authors:** Martina Laczó, Jan M. Wiener, Jana Kalinova, Veronika Matuskova, Martin Vyhnalek, Jakub Hort, Jan Laczó

**Affiliations:** 1 Memory Clinic, Department of Neurology, Charles University, Second Faculty of Medicine, Motol University Hospital, 150 06 Prague, Czech Republic; martina.laczo@gmail.com (M.L.); kalinja@email.cz (J.K.); veronika.matuskova@lfmotol.cuni.cz (V.M.); martin.vyhnalek@lfmotol.cuni.cz (M.V.); jakub.hort@lfmotol.cuni.cz (J.H.); 2 International Clinical Research Center, St. Anne’s University Hospital Brno, 656 91 Brno, Czech Republic; 3 Department of Psychology, Ageing and Dementia Research Centre, Bournemouth University, Poole BH12 5BB, UK; jwiener@bournemouth.ac.uk

**Keywords:** mild cognitive impairment, Alzheimer’s disease, spatial navigation, route learning, wayfinding, perspective taking, navigation strategies, visuospatial functions

## Abstract

Age-related spatial navigation decline is more pronounced in patients with mild cognitive impairment (MCI) and Alzheimer’s disease (AD) dementia. We used a realistic-looking virtual navigation test suite to analyze different aspects of visuospatial processing in typical and atypical aging. A total of 219 older adults were recruited from the Czech Brain Aging Study cohort. Cognitively normal older adults (CN; *n* = 78), patients with amnestic MCI (*n* = 75), and those with mild AD dementia (*n* = 66) underwent three navigational tasks, cognitive assessment, and brain MRI. Route learning and wayfinding/perspective-taking tasks distinguished the groups as performance and learning declined and specific visuospatial strategies were less utilized with increasing cognitive impairment. Increased perspective shift and utilization of non-specific strategies were associated with worse task performance across the groups. Primacy and recency effects were observed across the groups in the route learning and the wayfinding/perspective-taking task, respectively. In addition, a primacy effect was present in the wayfinding/perspective-taking task in the CN older adults. More effective spatial navigation was associated with better memory and executive functions. The results demonstrate that a realistic and ecologically valid spatial navigation test suite can reveal different aspects of visuospatial processing in typical and atypical aging.

## 1. Introduction

Spatial navigation is a complex cognitive ability important in everyday life for moving in both familiar and unfamiliar environments. Successful spatial navigation requires a combination of different navigation strategies. One of them is route learning (i.e., response learning), which entails creating a sequence of body movements (e.g., remembering right, left, right) or creating a series of stimulus–response associations when remembering associations of direction change with a specific landmark (e.g., “Turn left at the shop”) [1]. This strategy is referred to as egocentric navigation and is primarily based on self-motion perception but can also exploit environmental cues by creating self-to-object relations [2]. Route learning is primarily supported by the parietal cortex and the caudate nucleus [3].

A more flexible navigation strategy is wayfinding (i.e., place learning) where a navigator creates a mental representation (i.e., “cognitive map”) of the environment based on landmarks that are used as orientation cues. This entails the recognition of object-to-object relationships, and this strategy is referred to as allocentric navigation [2]. Wayfinding is primarily supported by the hippocampus and related medial temporal lobe structures [4].

Route learning and wayfinding are supported by different brain regions and are used in parallel and complement each other when navigating in the environment [5]. While the environment is typically perceived and encoded from the navigator’s perspective during navigation, successful navigation requires that the travelled route is recalled from a different direction, for example, when retracing a route (i.e., when navigating from the destination back to the starting place of a route). The flexibility to imagine scenes and environments from different viewpoints is referred to as perspective taking [2]. Perspective taking is supported by the parietal and temporal cortex [3].

Aging is associated with declines in spatial navigation abilities as documented by numerous studies [6,7]. Age-related spatial navigation declines are more pronounced in wayfinding than route learning, which is typically preserved for longer [6,8,9,10]. This decline could be a result of mild age-related functional and structural changes in the hippocampus and related medial temporal lobe structures [11,12]. Consequently, aging leads to an increasing preference for the route-learning strategy likely due to compensatory recruitment of extrahippocampal navigation strategies [8,13]. 

Alzheimer’s disease (AD) is characterized by profound spatial navigation impairment. This is likely to be a consequence of severe functional and structural changes in the brain regions that support spatial navigation, especially the hippocampus [14], the entorhinal cortex [15], and the parietal cortex [16], which are present in the early stages of the disease. AD is associated with route learning and wayfinding deficits. Wayfinding deficits in patients with mild cognitive impairment (MCI) and mild dementia were described in real-world environments when navigating in a hospital setting [17] and an enclosed circular arena [18], and in virtual environments when navigating in a virtual hallway complex [19]. 

In AD, wayfinding deficits are associated with smaller volumes of the hippocampus, entorhinal cortex, and basal forebrain [20,21,22]. Route-learning deficits are also present in AD [23] reflecting typical changes in the parietal cortex [24] and caudate nucleus [25]. Route-learning strategy preference increases over wayfinding with the progression of AD and reflects the recruitment of extrahippocampal navigation strategies [26]. In addition, the ability to imagine scenes during navigation from different spatial perspectives (viewpoint) (i.e., perspective taking) declines with age [27]. This deficit, which can be detected by virtual and paper-based tests, is more pronounced in AD [28]. 

In general, spatial navigation, especially wayfinding [6,8] and perspective taking [27], declines with aging. However, AD is associated with a greater spatial navigation decline including wayfinding, perspective taking, and route-learning, which is preserved in normal aging, and changes in preference of navigational strategies. These differences in profiles of spatial navigation dysfunctions indicate that spatial navigation assessment could be a useful tool to differentiate normal aging from the early stages of AD. 

Visuospatial functions are important for all types of spatial navigation as visuospatial perception and processing allow the navigator to locate relevant stimuli or landmarks in the environment, to make spatial judgments, and to visually discriminate different scenes [29]. Visuospatial perception and spatial information processing decline in older age [30,31]. Patients with AD experience even greater disruption of visuospatial functions [32,33]. Specifically, AD is associated with altered scene exploration and reduced utilization of important visual landmarks in the environment, which can cause patients to overlook landmarks critical for navigation [34] and may thus contribute to spatial navigation impairments. Visuospatial processing may influence the selection of a navigation strategy because the strategy choice depends on the perception of visual information in the environment [35,36]. Specifically, in the route-learning task, participants prefer using visual landmarks rather than geometric information to navigate in the environment [35]. Furthermore, spatial navigation is more accurate in virtual environments that feature visual landmarks [35,36]. Collectively, these studies demonstrate that visual input is highly important for strategy selection and successful spatial navigation.

Spatial navigation assessment should be ideally performed using an ecologically valid test that is easy to explain and where participants navigate in a realistic-looking environment. When using realistic-looking environments, it is possible to mimic real-life navigation and thus provide the most accurate measure of everyday-life navigation abilities [37]. The Navigation Test Suite (https://osf.io/mx52y/, accessed on 16 March 2017) designed by [38] consists of three spatial tasks in a realistic-looking virtual city: A route-repetition task evaluating route learning, and route-retracing and directional-approach tasks evaluating wayfinding, which can be also solved using perspective taking. The Navigation Test Suite was previously used to assess the effects of normal aging on spatial navigation [38]. The young participants outperformed the older participants in all three tasks (i.e., route-repetition, route-retracing, and directional-approach tasks). Specifically, older participants’ performance improved across the sessions in the route-repetition task but not in the route-retracing task. The young participants’ performance, in contrast, improved in both tasks. In the directional-approach task, where participants had to recall the street from which they originally approached an intersection when approaching it from a viewpoint they had not experienced before, the older adults showed a greater decline in performance than the young participants when the perspective shift between encoding and retrieval was higher.

We built on previous research using the Navigation Test Suite [38] and aimed to (1) assess differences in spatial navigation performance between cognitively normal (CN) older adults and patients with amnestic MCI (aMCI) and mild AD dementia, (2) evaluate differences in navigation strategy selection and the effect of strategy selection on spatial navigation performance, (3) analyze learning across the experimental sessions and the effect of perspective shift on spatial navigation performance, and (4) analyze serial position performance, which refers to primacy and recency effects, which means that information perceived at the beginning (primacy effect) and at the end (recency effect) are remembered better than information in the middle of a route. 

We hypothesized that: (1) the participants with aMCI and, even more, the participants with mild AD dementia would have less accurate spatial navigation performance in the route-learning, wayfinding, and perspective-taking task compared to the CN older adults; (2) the participants with aMCI and mild AD dementia would more often experience difficulties with creating an effective navigation strategy, which would lead to worse spatial navigation performance, (3) learning across the experimental sessions would be present in the CN group, less so in the aMCI group, and expressed the least in the mild AD dementia group, and (4) the serial position would be present differently in the CN, aMCI and mild AD dementia groups.

The novelty of this study is based on the fact that it combines three different spatial navigation tasks (i.e., route-learning, route-retracing, and wayfinding/perspective taking) in one ecologically valid spatial navigation paradigm to comprehensively assess differences between typical and atypical aging. Next, our study brings new information about differences in the selection and use of navigation strategies in CN older adults and patients with aMCI and mild AD dementia and their associations with spatial navigation performance. Further, our study sheds new light on the serial position effect in spatial navigation. Up to now, the serial position effect has been assessed primarily in memory tests where a characteristic pattern of the serial position effect is preserved in older adults even when overall memory decreases [39], whereas patients with MCI typically show a primacy effect lower than the recency effect [40]. A recent study showed primacy and recency effects in route learning in young and older adults [41]. To the best of our knowledge, our study is the first to analyze primacy and recency effects in spatial navigation in patients with MCI and AD dementia. The Navigation Test Suite also allows us to assess the effect of spatial navigation learning and the effect of perspective shift on spatial navigation performance, which have been reported previously [18,28] but not in an ecologically valid and realistic-looking large-scale virtual environment.

## 2. Methods

### 2.1. Participants

A total of 219 participants were recruited from the Czech Brain Aging Study cohort [42] at the Memory Clinic of the Charles University, Second Faculty of Medicine and Motol University Hospital in Prague, Czech Republic, and signed an informed consent approved by the local ethics committee [43]. The participants with cognitive deficit were referred to the Memory Clinic by general practitioners and neurologists for memory complaints reported by themselves and/or by their relatives. CN older participants were recruited from the University of the Third Age, senior centers (e.g., the Elpida center), and relatives of the participants and hospital staff [44].

All participants underwent clinical and laboratory evaluations, comprehensive cognitive assessment, brain magnetic resonance imaging (MRI), and completed the Navigation Test Suite. The participants were classified into three groups: mild AD dementia, aMCI, and CN older adults based on clinical criteria, the information provided by the participants and their informants, and cognitive assessment. The demographic and cognitive characteristics are listed in Table 1.

(1) Participants with mild AD dementia (*n* = 66) met the clinical criteria for AD dementia [45] with evidence of progressive cognitive impairment in at least two cognitive domains including memory (i.e., score lower than 1.5 standard deviations (SDs) below the age- and education-adjusted norms in any memory test and in at least one other non-memory cognitive test) [26] and significant impairment in activities of daily living and had hippocampal atrophy (i.e., pathological medial temporal lobe atrophy score) [46].
(2) Participants with aMCI (*n* = 75) met the clinical criteria for aMCI [47] including memory complaints, evidence of memory impairment (i.e., score lower than 1.5 SDs below the age- and education-adjusted norms in any memory test) [26], generally intact activities of daily living, and the absence of dementia.
(3) CN participants (*n* = 78) did not report any cognitive complaints and had cognitive performance within the normal range (i.e., score higher than 1.5 SDs below the age- and education-adjusted norms in any cognitive test). In addition, they had no evidence of hippocampal atrophy on MRI (i.e., normal medial temporal lobe atrophy score) [46] and did not have a family history of neurodegenerative diseases in first-degree relatives. These stringent criteria were applied to minimize the possibility of including participants with preclinical and early clinical neurodegenerative diseases.

Participants with depressive symptoms (≥6 points on the 15-item Geriatric Depression Scale (GDS-15)), anxiety (≥10 points on the Beck Anxiety Inventory (BAI)), low visual acuity (<20/40 (corrected) on visual acuity tests), moderate to severe white matter vascular lesions on MRI (Fazekas score >2 points), and other primary neurological (history of stroke, Parkinson’s disease, epilepsy, brain tumor) or psychiatric disorders [44] and those who did not successfully complete the training in the Navigation Test Suite were not included in the study.

### 2.2. The Navigation Test Suite

**Figure 1:**
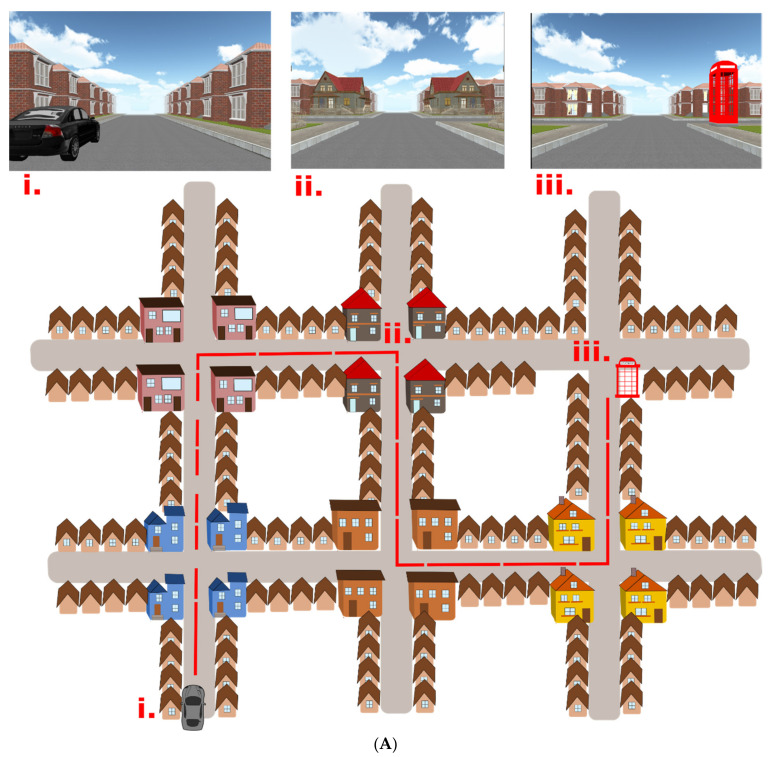

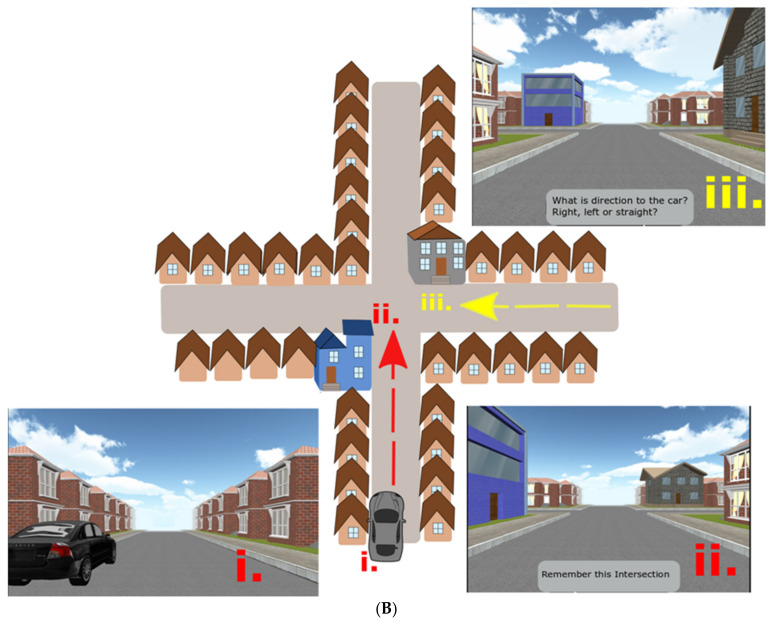
(**A**) The Navigation Test Suite with schematic aerial view and corresponding screenshots from the route-repetition and the route-retracing tasks. Three points on the map are labeled: (i.) The start location next to the car. (ii.) One of the intersections along the route with gray houses at the corners of the intersection. (iii.) The end of the route where the telephone box is present. In the route-repetition task, the participants were passively transported through the city from the car to the telephone box during the encoding phase and in the test phase the participants had to reproduce the same route. The route-retracing task was identical to the route-repetition task with the exception that participants in the test phase had to find their way back from the telephone box to the car. The order of intersections and houses at each intersection had a different design in each of these two tasks. (**B**) The Navigation Test Suite with schematic aerial view and corresponding screenshots from the directional-approach task: (i.) Participants started the task next to the car. (ii.) The encoding phase, where participants were passively transported towards one of the intersections featuring two unique houses. Participants had to remember where the car was parked. (iii.) The test phase, where participants approached the intersection from a different direction (here from east) and had to indicate direction to the car.

We used the Navigation test suite, which is described in detail in [38]. The Navigation Test Suite consists of three navigation tasks: The route-repetition task, the route-retracing task, and the directional-approach task. The Navigation Test Suite uses a virtual environment that consists of streets and four-way intersections with residential houses along the streets. The houses bordering the streets are all identical, except the unique houses (i.e., distinct landmarks) that are located at each intersection (explained in more detail below, Figure 1A,B). Participants can always see only one intersection at any time, because the other more distant intersections are concealed in white fog.

(I) Route-repetition task

In the encoding phase, the participants were positioned in a street next to a black car. They were then passively transported along a route featuring five intersections with one right turn, three left turns, and one straight movement. The route then stopped at a red phone box. Each intersection featured four identical houses at the four corners. Different intersections featured different houses, so that each intersection had a unique appearance. Participants were instructed to remember the route (Figure 1A).

In the test phase, the participants were asked to reproduce the same route from the car to the phone box. Participants were passively transported towards each of the intersections where they were stopped 20 m before the center of the intersections and prompted to verbally indicate the direction in which the route continued. The examiner pressed a corresponding arrow key, and the participants were passively transported to the center of the intersection facing the street, which led to the following intersection. Thus, participants did not receive feedback. The task was composed of three experimental sessions to assess learning.

(II) Route-retracing task

The encoding phase was similar to the route-repetition task, but the route was different and featured different houses at intersections (Figure 1A). In the test phase, the participants had to navigate in the opposite direction compared to the encoding phase, i.e., from the end point of the route (the telephone box) back to the start (the black car).

The route-repetition and route-retracing tasks consisted of 3 identical consecutive sessions and each session contained a route with 5 intersections. After completing each task, participants were asked to describe the strategy they used to solve the task. The reported strategies were then classified into three groups: (1) Sequence-of-directions, remembering a sequence of movement directions (e.g., left, right, straight, left, right) regardless of landmarks, (2) stimulus–response, creating associations between the places or landmarks with the movement direction (e.g., turn left at the intersection with blue houses), and (3) non-specific, such that participants reported inability to devise any strategy, or they used objects that could not be used as landmarks (e.g., observing clouds).

(III) Directional-approach task

This task tested participants’ ability to encode the configuration of houses (landmarks) at an intersection and assessed wayfinding and perspective-taking abilities [48,49]. This task consisted of 15 independent trials. Each trial began with an encoding phase, in which participants were positioned in a street next to a black car from where they were passively transported toward a single intersection, which featured two unique houses (i.e., landmarks) at diagonally opposite corners of the intersection. The movement stopped 20 m before the center of the intersections, so both unique houses were in view. Two other houses at the corners of the intersection were identical with the other houses along the street. The participants’ task was to memorize in which street the car was parked. Each of the 15 trials featured a different combination of unique houses at the corners of the intersections. 

In the test phase, participants were passively transported toward the same intersection, but from one of the other streets. They were then asked to indicate the direction in which the car was parked (i.e., to indicate the street from which they originally approached the intersection). The movement stopped again 20 m before the center of the intersection, such that the unique houses could be seen. 

The car was always parked in the street to the south of the intersection (Figure 1B). In the test phase, participants approached the intersection from the west, east, or north street. The participants were not aware of these cardinal directions in the experiment, but the information about these cardinal directions was used in the analysis. Participants were required to perform perspective shifts to align the view during the test phase with that during the perspective in the encoding phase. The perspective shift was 90° when approaching the intersection from the west or east and 180° when approaching from the north. As opposed to the previous route-repetition and route-retracing tasks, the directional-approach task did not require participants to learn a route with multiple decision points.

Participants were asked about the navigation strategy after the Directional-approach task. Reported strategies were classified into three groups: (1) Unique houses, remembering the position of one or two unique houses at the intersections, (2) more houses, remembering three, all four, or non-unique houses, and (3) non-specific, (e.g., guessing, remembering grass, which was same at all intersections).

In all three tasks, participants indicated their responses verbally and the experimenter pressed the corresponding arrow key on the keyboard. A correct response at each of the intersections was counted as one point. Regarding the strategy analysis, participants were asked “How did you find the way?” at the end of each task. This was a very open question allowing the participant to describe their strategy in their own words. In the case when the response was not entirely clear, the participants were given another question for clarification “Could you please describe this in greater detail?”. The participants were never asked suggestive questions. The examiner wrote down the participants’ responses. Categories of navigation strategies were created retrospectively (when all responses were available) based on the given responses. Categorization was performed by two experienced examiners blinded to all other information about participants and their performance, each of the examiners classified the responses independently, and questionable responses were discussed with the supervisor.

Prior to the testing, all participants completed familiarization training consisting of shorter versions of all three tasks (a three-intersection path for the route-repetition and route-retracing tasks and two separate intersections for the directional-approach task) [38]. All CN participants and participants with aMCI and 51 out of 66 participants with mild AD dementia completed the training and all three tasks of the Navigation Test Suite. The participants who did not understand the training were not included in the study.

### 2.3. Cognitive Assessment

The cognitive assessment included the following tests: (1) Verbal memory measured with the Rey Auditory Verbal Learning Test—trials 1–5 and 30-min Delayed Recall trial and the Logical Memory I—Immediate and 20-min Delayed Recall conditions; (2) non-verbal memory measured with the Rey–Osterrieth Complex Figure Test—Recall condition after 3 min; (3) visuospatial function measured with the Rey–Osterrieth Complex Figure Test—Copy condition and the Clock Drawing Test; (4) executive function measured with the Trail Making Test B, the Prague Stroop Test—colors, and the Controlled Oral Word Association Test (Czech version with letters N, K, and P); (5) attention and working memory measured with the Forward and Backward Digit Spans and the Trail Making Test A; and (6) language measured with the Boston Naming Test (30-item version) and the Category Fluency test (Animals). The Mini-Mental State Examination (MMSE) was administered to measure global cognitive function. The GDS-15 and BAI were used to assess depressive symptoms and anxiety among participants. Group-wise neuropsychological characteristics are listed in Table 1.

### 2.4. Data Analysis

For continuous demographic and cognitive variables, a one-way analysis of variance (ANOVA) with post hoc Sidak’s test was used. For changes in proportion (gender and selected strategies), a χ^2^ test was used. Pearson’s correlation with Holm–Bonferroni correction for multiple comparisons was used to assess associations between navigational tests and cognitive performance in each group. A two-way ANOVA and general linear models using the same data but focusing on different aspects of spatial navigation were performed for each task of the Navigation Test Suite and are described in detail below. 

In the route-repetition and route-retracing tasks, a repeated-measures analysis of covariance (RM-ANCOVA) with the session (1st, 2nd, and 3rd) as the within-subjects factor and the group (CN, aMCI, and mild AD dementia) as the between-subjects factor was used to assess spatial navigation performance. Performance was measured as the percentage of trials in which participants provided a correct response. The analyses were controlled for age (mean-centered), years of education (mean-centered) and gender. Where applicable, the Greenhouse–Geisser and Huynd–Feldt corrections were used to correct for the violation of sphericity when epsilon was ≤0.75 and >0.75, respectively. The planned polynomial contrasts were used to assess the effect of the session. The post hoc Sidak’s test was used to assess differences between the individual groups and sessions. A one-sample t-test was used to assess differences from chance performance (i.e., 33.33%) for each group in each task and within each session. A two-way ANOVA with post hoc Sidak’s test was used to evaluate the effect of group and reported strategy on spatial navigation performance. An RM-ANOVA with the order of intersections (1st, 2nd, 3rd, 4th, and 5th) as the within-subjects factor and group as the between-subjects factor was used to assess the serial position performance. Where applicable, the Greenhouse–Geisser and Huynd–Feldt corrections were used to correct for the violation of sphericity. The planned polynomial contrasts were used to assess the effect of the order of intersections and the post hoc Sidak’s test was used to assess differences between the individual groups and orders of intersections.

In the directional-approach task, an RM-ANCOVA with the approach direction (west, north, and east) as the within-subjects factor and group (CN, aMCI, and mild AD dementia) as the between-subjects factor was used to assess spatial navigation performance. Again, the analysis was controlled for age (mean-centered), education (mean-centered), and gender, the Greenhouse–Geisser and Huynd–Feldt corrections were used to correct for the violation of sphericity (where applicable), the planned polynomial contrasts were used to assess the effect of approach direction, and the post hoc Sidak’s test was used to assess differences between the individual groups and specific approach directions. A one-sample t-test was used to assess differences from chance performance (i.e., 33.33%) for each group in the task and within each approach direction. A two-way ANOVA with post hoc Sidak’s test was used to evaluate the effect of group and reported strategy on spatial navigation performance.

## 3. Results

### 3.1. Demographic Characteristics

The demographic characteristics are presented in detail in Table 1. The CN group was younger than the aMCI and mild AD dementia groups (both *p* ≤ 0.020) and the aMCI group was younger than the mild AD dementia group (*p* = 0.001). The CN group was more educated that the aMCI and mild AD dementia groups (both *p* ≤ 0.028). There were more women in the CN and mild AD dementia groups than in the aMCI group (71.8% and 63.6% vs. 44.0%) [*χ*^2^(2) = 43.21, *p* = 0.002]. As expected, the CN group had higher MMSE scores (both *p* < 0.001) and showed better cognitive performance (both *p* ≤ 0.025) than the aMCI and mild AD dementia groups. There were no differences in depressive and anxiety symptoms between the groups (all *p* ≥ 0.133).

### 3.2. Route-Repetition Task

**Figure 2:**
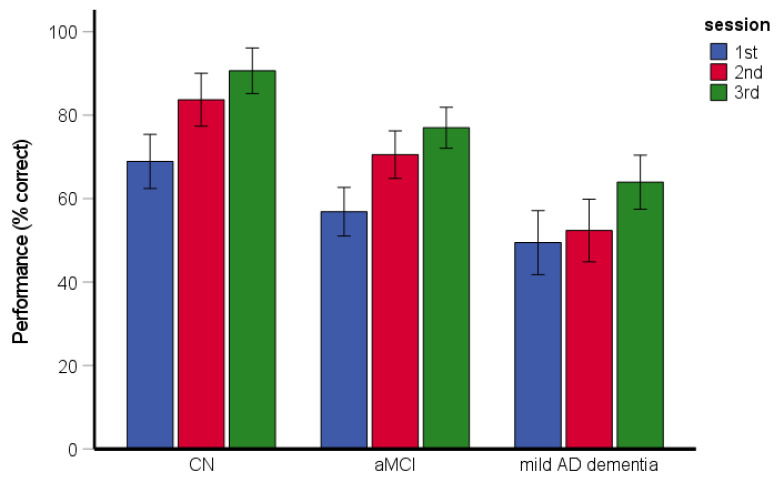
Route-repetition task—spatial navigation performance and learning across the three experimental sessions. CN, cognitively normal; aMCI, amnestic mild cognitive impairment; mild AD dementia, mild Alzheimer’s disease dementia.

The RM-ANCOVA assessing spatial navigation performance and learning across the sessions revealed significant main effects of group [*F*(2, 193) = 24.12, *p* < 0.001, η_p_^2^ = 0.20] and session [*F*(2, 386) = 41.69, *p* < 0.001, η_p_^2^ = 0.18] (Figure 2). Specifically, the CN group performed better than the aMCI and mild AD dementia groups (both *p* < 0.001) and the aMCI group performed better than the mild AD dementia group (*p* = 0.001). Performance in the second experimental session was better than that in the first experimental session (*p* < 0.001) and performance in the third experimental session was better than that in the first and second experimental sessions (both *p* < 0.001). The interaction between group and experimental session was not significant [*F*(4, 386) = 1.29, *p* = 0.273, η_p_^2^ = 0.01]. All groups performed above chance level in the task overall and within each session (CN group: [all *t*(77) ≥ 12.76, *p* < 0.001]; aMCI group: [all *t*(74) ≥ 7.77, *p* < 0.001]; mild AD dementia group: [all *t*(50) ≥ 4.52, *p* < 0.001]). The effect of age [*F*(1, 193) = 4.77, *p* = 0.030, η_p_^2^ = 0.24] and education [*F*(1, 193) = 4.50, *p* = 0.035, η_p_^2^ = 0.23] was significant. The effect of gender [*F*(1, 193) = 0.73, *p* = 0.395, η_p_^2^ = 0.00] and the gender-by-group interaction [*F*(2, 193) = 0.80, *p* = 0.451, η_p_^2^ = 0.01] were not significant.

The χ^2^ test revealed the differences between the groups in reported strategies [*χ^2^*(4) = 43.21, *p* < 0.001, Cramer’s V = 0.33]. Specifically, the CN participants and participants with aMCI reported the use of the non-specific strategy less frequently than the stimulus–response and the sequence-of-directions strategies (75.3% sequence-of-directions, 23.4% stimulus–response, 1.3% non-specific and 80.3% sequence-of-directions, 14.1% stimulus–response, 5.6% non-specific, respectively), while the participants with mild AD dementia reported the use of the non-specific and sequence-of-directions strategies with similar frequency (44.9% sequence-of-directions, 18.4% stimulus–response, 36.7% non-specific).

**Figure 3:**
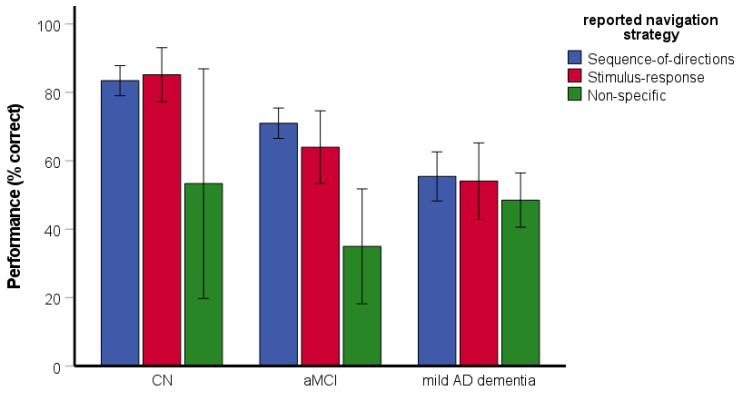
Route-repetition task—the effect of reported strategy on spatial navigation performance. CN, cognitively normal; aMCI, amnestic mild cognitive impairment; mild AD dementia, mild Alzheimer’s disease dementia.

The two-way ANOVA assessing the effect of reported strategy on spatial navigation performance revealed significant main effects of group [*F*(2, 188) = 5.50, *p* = 0.005, η_p_^2^ = 0.06] and reported strategy [*F*(2, 188) = 6.68, *p* = 0.002, η_p_^2^ = 0.07] (Figure 3). Specifically, the CN group performed better than the aMCI (*p* = 0.035) and mild AD dementia (*p* = 0.003) groups. The aMCI group did not differ from the mild AD dementia group (*p* = 0.735). The participants reporting the use of the non-specific strategy had worse performance than those reporting the use of the sequence-of-directions (*p* = 0.001) and the stimulus–response (*p* = 0.006) strategies. The performance did not differ between participants reporting the use of the sequence-of-directions and the stimulus–response strategies (*p* = 0.881). The interaction between group and reported strategy was significant [*F*(4, 188) = 2.52, *p* = 0.043, η_p_^2^ = 0.05]. Specifically, the participants with aMCI reporting the use of the non-specific strategy had worse performance than those reporting the use of the sequence-of-directions (*p* < 0.001) and stimulus–response (*p* = 0.004) strategies but the participants with mild AD dementia did not differ in performance with respect to the reported strategy (all *p* ≥ 0.201). As there was only one CN participant reporting the use of the non-specific strategy, a post-hoc one-sample t-test was used. The test revealed that the CN participant reporting the use of the non-specific strategy had worse performance than those reporting the use of the sequence-of-directions [*t*(57) = 14.04, *p* < 0.001] and stimulus–response [*t*(17) = 9.06, *p* < 0.001] strategies.

**Figure 4:**
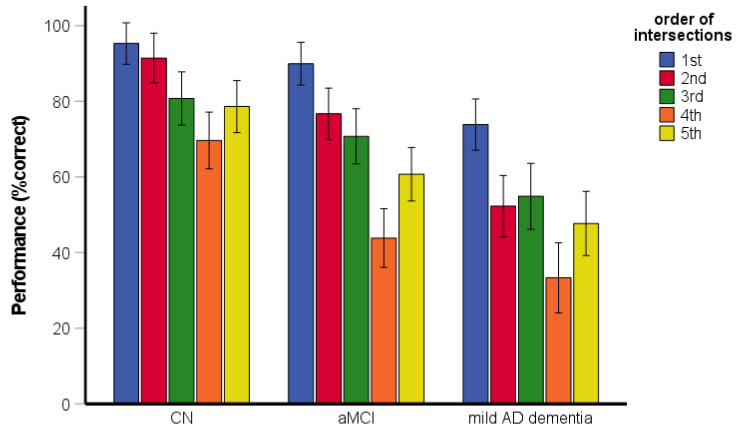
Route-repetition task—the serial position effect. CN, cognitively normal; aMCI, amnestic mild cognitive impairment; mild AD dementia, mild Alzheimer’s disease dementia.

The RM-ANOVA assessing the serial position performance revealed significant main effects of group [*F*(2, 199) = 47.06, *p* < 0.001, η_p_^2^ = 0.32] and the order of intersections [*F*(3.68, 731.26) = 50.16, *p* < 0.001, η_p_^2^ = 0.20] (Figure 4). Specifically, the CN group performed better than the aMCI and mild AD dementia groups (both *p* < 0.001) and the aMCI group performed better than the mild AD dementia group (*p* < 0.001). Performance for the first intersection was better than that for the third (i.e., middle) intersection (*p* < 0.001) and the fifth (i.e., last) intersection (*p* < 0.001). Performance for the fifth intersection did not differ from that for the third intersection (*p* = 0.202). The interaction between group and order of intersections was significant [*F*(7.35, 731.26) = 2.20, *p* = 0.030, η_p_^2^ = 0.02]. Specifically, the aMCI group performed worse for the fifth intersection than for the third intersection (*p* = 0.030) but performance for the fifth intersection did not differ from that for the third intersection in the CN (*p* = 0.630) and mild AD dementia (*p* = 0.191) groups. In all groups, the performance for the first intersection was better than for the third (all *p* < 0.001) and fifth (all *p* < 0.001) intersections.

### 3.3. Route-Retracing Task

**Figure 5:**
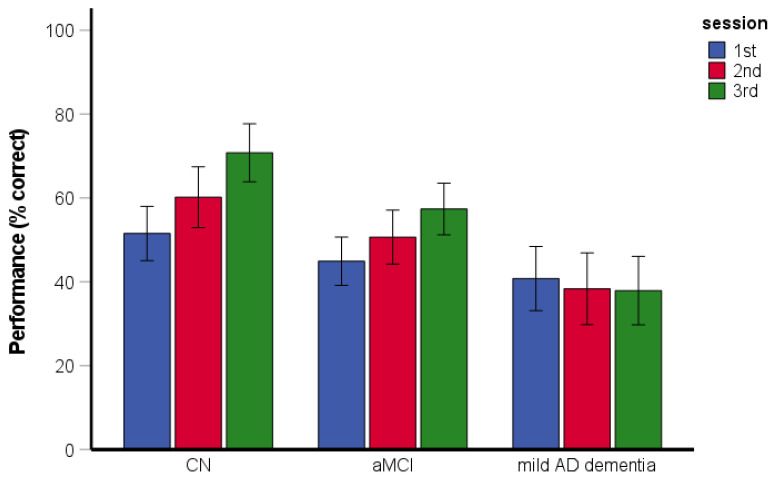
Route-retracing task—spatial navigation performance and learning across the sessions. CN, cognitively normal; aMCI, amnestic mild cognitive impairment; mild AD dementia, mild Alzheimer’s disease dementia.

The RM-ANCOVA assessing spatial navigation performance and learning across the sessions revealed significant main effects of group [*F*(2, 194) = 14.99, *p* < 0.001, η_p_^2^ = 0.13] and session [*F*(2, 388) = 7.79, *p* < 0.001, η_p_^2^ = 0.04] (Figure 5). Specifically, the CN group performed better than the aMCI (*p* = 0.010) and mild AD dementia (*p* < 0.001) groups and the aMCI group performed better than the mild AD dementia group (*p* = 0.003). Performance in the third experimental session was better than that in the first (*p* = 0.001) and second (*p* = 0.044) experimental sessions. Performance in the second experimental session did not differ from that in the first experimental session (*p* = 0.290). The interaction between group and experimental session was significant [*F*(4, 388) = 2.62, *p* = 0.035, η_p_^2^ = 0.03]. This interaction was driven by a significant increase in performance in the CN group across all sessions (session 1 vs. session 2: *p* = 0.046; session 1 vs. session 3: *p* < 0.001; session 2 vs. session 3: *p* = 0.009), whereas performance in the aMCI group improved significantly only between the first and the third session (session 1 vs. session 3: *p* = 0.002; session 1 vs. session 2: *p* = 0.137; session 2 vs. session 3: *p* = 0.061), and performance in the mild AD dementia group did not significantly improve across the sessions (all *p* ≥ 0.592). The CN and aMCI groups performed above chance level (CN group: [all *t*(77) ≥ 6.92, *p* < 0.001]; aMCI group: [all *t*(73) ≥ 3.88, *p* < 0.001]), whereas performance (overall and within each session) in the mild AD dementia group did not differ from chance level [all *t*(50) ≤ 1.39, *p* ≥ 0.170]. The effect of age [*F*(1, 194) = 4.47, *p* = 0.036, η_p_^2^ = 0.23] and education [*F*(1, 194) = 3.95, *p* = 0.048, η_p_^2^ = 0.02] was significant. The effect of gender [*F*(1, 194) = 0.97, *p* = 0.326, η_p_^2^ = 0.01] and the gender-by-group interaction [*F*(2, 194) = 0.37, *p* = 0.692, η_p_^2^ = 0.00] were not significant.

The χ^2^ test revealed differences between the groups in the reported strategies [*χ*^2^(4) = 27.70, *p* < 0.001, Cramer’s V = 0.27]. Specifically, the CN participants reported the use of the non-specific strategy less frequently than the stimulus–response and the sequence-of-directions strategies (70.1% sequence-of-directions, 23.4% stimulus–response, 6.5% non-specific) and the participants with aMCI reported the use of the non-specific strategy less frequently than the sequence-of directions strategy (68.6% sequence-of-directions, 17.1% stimulus–response, 14.3% non-specific), while the participants with mild AD dementia reported the use of the non-specific and the sequence-of-directions strategies with similar frequency (36.7% sequence-of-directions, 22.4% stimulus–response, 40.8% non-specific).

**Figure 6:**
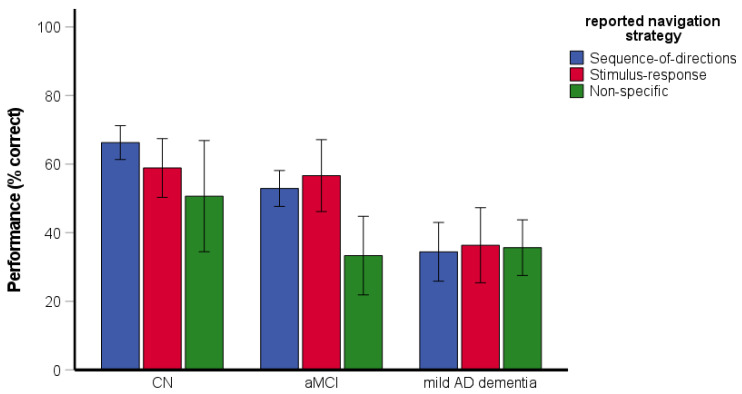
Route-retracing task—the effect of reported strategy on spatial navigation performance. CN, cognitively normal; aMCI, amnestic mild cognitive impairment; mild AD dementia, mild Alzheimer’s disease dementia.

The two-way ANOVA assessing the effect of the reported strategy on spatial navigation performance revealed significant main effects of group [*F*(2, 187) = 15.39, *p* < 0.001, η_p_^2^ = 0.14] and reported strategy [*F*(2, 187) = 3.99, *p* = 0.020, η_p_^2^ = 0.04] (Figure 6). Specifically, the CN group performed better than the aMCI (*p* = 0.031) and mild AD dementia (*p* < 0.001) groups and the aMCI group performed better than the mild AD dementia group (*p* = 0.006). The participants reporting the use of the non-specific strategy showed worse performance than those reporting the use of the sequence-of-directions strategy (*p* = 0.018). Differences in performance between participants reporting the use of the non-specific strategy and the stimulus–response strategy did not reach statistical significance (*p* = 0.065). The performance did not differ between participants reporting the use of the sequence-of-directions and the stimulus–response strategies (*p* = 0.998). The interaction between group and reported strategy did not reach statistical significance [*F*(4, 187) = 2.23, *p* = 0.068, η_p_^2^ = 0.05].

**Figure 7:**
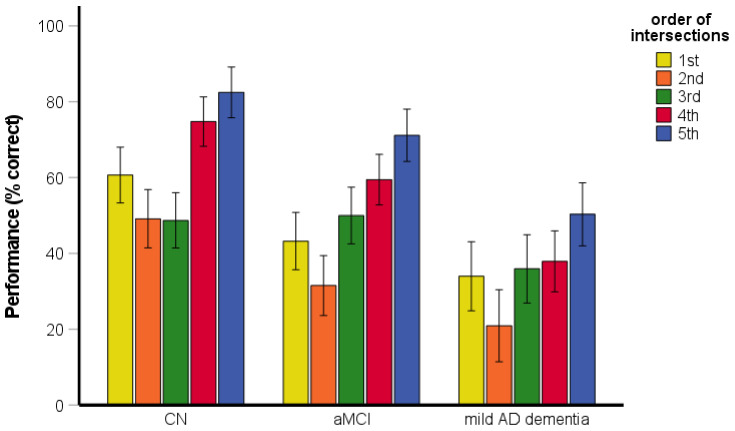
Route-retracing task—the serial position effect. Intersections in the route-retracing task were recalled in reverse order than in which they were presented during the encoding phase. Order of intersections refers to the order in the test phase (i.e., the 1st intersection that was first recalled in the test phase was the last encoded during the encoding phase). CN, cognitively normal; aMCI, amnestic mild cognitive impairment; mild AD dementia, mild Alzheimer’s disease dementia.

The RM-ANOVA assessing the serial position performance revealed significant main effects of group [*F*(2, 200) = 32.54, *p* < 0.001, η_p_^2^ = 0.25] and the order of intersections [*F*(3.82, 763.03) = 40.26, *p* < 0.001, η_p_^2^ = 0.17] (Figure 7). Specifically, the CN group performed better than the aMCI and mild AD dementia groups (*p* < 0.001) and the aMCI group performed better than the mild AD dementia group (*p* < 0.001). Performance for the fifth intersection based on the order of intersections in the encoding phase (i.e., the first intersection in the test phase) was better than that for the third (i.e., middle) intersection (*p* < 0.001) and the first intersection (i.e., the fifth intersection in the test phase) (*p* < 0.001). Performance for the first intersection did not differ from that for the third intersection (*p* = 1.000). The interaction between group and order of intersections was significant [*F*(7.63, 763.03) = 2.40, *p* = 0.016, η_p_^2^ = 0.02]. Specifically, performance for the first intersection was better than for the third intersection in the CN group (*p* = 0.014) but performance for the first intersection did not differ from that for the third intersection in the aMCI (*p* = 0.175) and mild AD dementia (*p* = 0.744) groups. Performance for the fifth intersection was better than for the third (all *p* ≤ 0.007) and first (all *p* ≤ 0.004) intersections in all groups.

### 3.4. Directional-Approach Task

**Figure 8:**
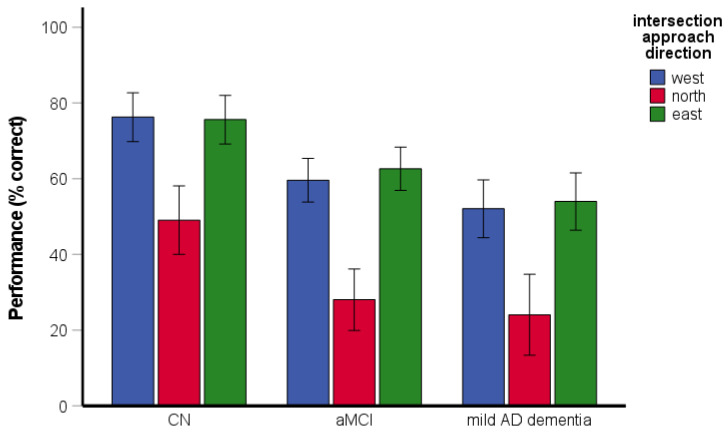
Directional-approach task—spatial navigation performance and the effect of approach direction. CN, cognitively normal; aMCI, amnestic mild cognitive impairment; mild AD dementia, mild Alzheimer’s disease dementia.

The RM-ANCOVA assessing spatial navigation performance and the effect of approach direction on navigational performance revealed significant main effects of group [*F*(2, 193) = 18.21, *p* < 0.001, η_p_^2^ = 0.16] and approach direction [*F*(1.82, 350.92) = 81.11, *p* < 0.001, η_p_^2^ = 0.30] (Figure 8). Specifically, the CN group performed better than the aMCI and the mild AD dementia groups (both *p* < 0.001) and the aMCI group did not differ from the mild AD dementia group (*p* = 0.222). Performance was worse for an approach from the north than for an approach from the east (*p* < 0.001) and the west (*p* < 0.001). Performance for the west and east approaches did not differ from each other (*p* = 0.875). The interaction between group and approach direction was not significant [*F*(3.64, 350.92) = 0.43, *p* = 0.772, η_p_^2^ = 0.00]. For an approach from the north, the CN group performed above chance level [*t*(77) = 3.05, *p* = 0.003], whereas performance in the aMCI group did not differ from chance level [*t*(72) = −1.07, *p* = 0.289] and the mild AD dementia group performed below chance level [*t*(50) = −4.13, *p* < 0.001]. All groups performed above chance level in the task overall and for approaches from the east and the west (CN group: [all *t*(77) ≥ 13.55, *p* < 0.001]; aMCI group: [all *t*(72) ≥ 6.87, *p* < 0.001]; mild AD dementia group: [all *t*(50) ≥ 2.79, *p* ≤ 0.008]). The effect of age was significant [*F*(1, 193) = 8.79, *p* = 0.003, η_p_^2^ = 0.04], while the effect of education was not significant [*F*(1, 193) = 1.02, *p* = 0.313, η_p_^2^ = 0.01]. The effect of gender was significant [*F*(1, 193) = 8.55, *p* = 0.004, η_p_^2^ = 0.04], where women generally had worse performance than men. The gender-by-group interaction [*F*(2, 193) = 0.98, *p* = 0.378, η_p_^2^ = 0.01] was not significant.

The χ^2^ test revealed differences between the groups in reported strategies [χ^2^(4) = 53.45, *p* < 0.001, Cramer’s V = 0.37]. Specifically, the CN participants reported the use of the unique-houses strategy more frequently than the more-houses and non-specific strategies (64.5% unique houses, 30.3% more houses, 5.3% non-specific), while the participants with aMCI reported the use of the unique-houses and more-houses strategies with similar frequency (52.9% unique houses, 44.3% more houses, 2.9% non-specific). The participants with mild AD dementia reported the use of the non-specific strategy more frequently than the unique-houses and the more-houses strategies (28.6% unique houses, 26.5% more houses, 44.9% non-specific).

**Figure 9:**
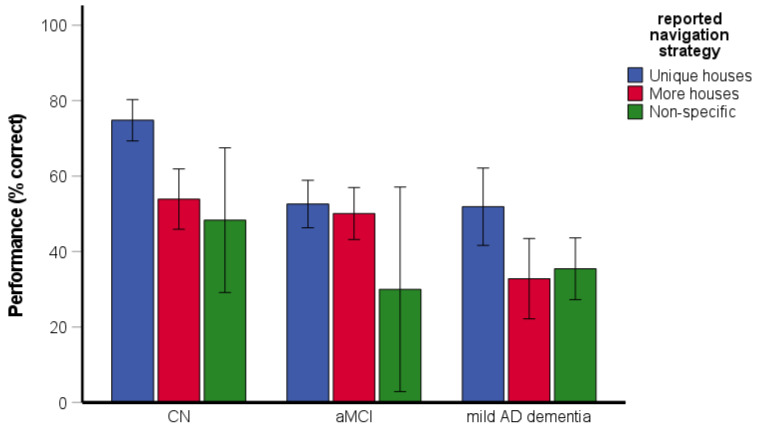
Directional-approach task—the effect of reported strategy on spatial navigation performance. CN, cognitively normal; aMCI, amnestic mild cognitive impairment; mild AD dementia, mild Alzheimer’s disease dementia.

The two-way ANOVA assessing the effect of reported strategy on spatial navigation performance revealed significant main effects of group [*F*(2, 186) = 8.60, *p* < 0.001, η_p_^2^ = 0.09] and reported strategy [*F*(2, 186) = 12.19, *p* < 0.001, η_p_^2^ = 0.12] (Figure 9). Specifically, the CN group performed better than the aMCI (*p* = 0.046) and the mild AD dementia (*p* < 0.001) groups and the aMCI group did not differ from the mild AD dementia group (*p* = 0.841). The participants reporting the use of the unique-houses strategy showed better performance than those reporting the use of the more-houses (*p* < 0.001) and non-specific (*p* = 0.002) strategies. Performance did not differ between the participants reporting the use of the more-houses and non-specific strategies (*p* = 0.534). The interaction between group and reported strategy did not reach statistical significance [*F*(4, 186) = 2.40, *p* = 0.052, η_p_^2^ = 0.05].

### 3.5. Correlations between Navigational Tasks and Cognitive Performance

Pearson’s correlation with Holm–Bonferroni correction for multiple comparisons revealed a significant correlation between the route-repetition, route-retracing, and directional-approach tasks in the aMCI group (r = 0.39–0.49, all *p* ≤ 0.001). The correlation was not significant or did not survive the adjustment for multiple comparisons in the CN group (r = 0.20–0.26, *p* = 0.075–0.022) and was not significant in the mild AD dementia group (r = −0.09–−0.19, all *p* ≥ 0.179). Because all three navigational tasks correlated with each other and to minimize a Type II error due to the correction for multiple comparisons, their scores were combined into a single total navigational score for correlational analysis with cognitive performance. In the analysis, the total navigational score correlated with verbal and non-verbal memory, executive and language functions in the CN group (r = 0.30–0.35, *p* = 0.002–0.007), verbal and non-verbal memory in the aMCI group (r = 0.23, *p* = 0.005 and r = 0.51, *p* < 0.001, respectively), and executive function in the mild AD dementia group (r = 0.47, *p* = 0.001).

## 4. Discussion

This study aimed to comprehensively explore spatial navigation in CN older adults and patients with aMCI and mild AD dementia. Specifically, we examined the differences in spatial navigation performance and navigation strategy selection, evaluated the effect of strategy selection on navigational performance, and analyzed learning across the experimental sessions, the effect of perspective shift on navigational performance, the serial position (i.e., primacy/recency) effect, and the associations between spatial navigation and cognitive performance. For this purpose, we used three navigational tasks in a realistic-looking virtual city addressing the ability to learn a route, the ability to retrace a recently travelled route, and the ability to learn and use a configuration of landmarks. Participants were interviewed about the use of navigation strategies to investigate visuospatial perception and its influence on spatial navigation performance.

In the route-repetition task, we observed route-learning deficits in aMCI patients that were even more pronounced in patients with mild AD dementia. This is in line with previous studies that reported route-learning impairment in MCI patients [24,50,51] and patients with AD [17,19,50,51,52] and a study showing more severe route-learning impairment in AD patients than those with MCI [50]. All groups showed learning across three experimental sessions. Learning in CN older adults in our study is in agreement with a previous study using the same experimental task that reported learning over the course of the experiment in older participants [38]. Learning in participants with a cognitive deficit that we have observed in the current study was not shown in previous studies using route-learning tasks in patients with aMCI [24] and AD [19]. It should be noted that these previous route-learning studies used longer routes with six and twelve intersections, respectively, compared to our route that consisted of five intersections. Our findings indicate that patients with aMCI and mild AD dementia are able to learn unfamiliar routes when the number of intersections does not exceed the spatial span of AD patients [53]. 

Participants most frequently reported that they remembered a sequence of turns regardless of landmarks (i.e., the sequence-of-directions strategy) and less frequently that they created associations between landmarks and movement directions (i.e., the stimulus–response strategy). Only very few of the CN older adults and aMCI patients reported that they did not devise any specific strategy (i.e., non-specific strategy). In contrast, the sequence-of-directions strategy and the non-specific strategy were the most frequently reported strategies in patients with mild AD dementia. None of the participants reported creating a cognitive map to solve this route-learning task. 

Route-learning performance was similar when using the sequence-of-direction strategy and the stimulus–response strategy. This is not surprising since both of these strategies are referred to as egocentric strategies because they rely on spatial information encoded in an egocentric reference frame [54]. The use of the non-specific strategy compared to adopting any of the egocentric strategies was associated with worse task performance, especially in CN older adults and aMCI patients. This finding is in line with a previous study that showed better route-learning performance in aMCI patients using a specific strategy compared to those using no specific strategy [24] and underlines the important role of the sequence-of-direction and stimulus–response strategies in route learning [1]. 

To the best of our knowledge, this is the first study analyzing serial position effects in a route-learning task in older adults with aMCI and mild AD dementia. We observed a primacy effect in all participant groups, where participants showed the best performance in the first intersection. Such a serial position effect has been reported in previous navigation studies [55,56]. A recent route-learning study showed the serial position effect for landmarks encountered during navigation with strong primacy and recency benefits in CN older adults [41]. In patients with MCI and AD dementia, list-learning tasks that are used to investigate serial position effects usually show a diminished primacy effect (for review see [57]), which was not observed in our navigational study. These discordant findings could be caused by different experimental protocols used in the current study and previous studies, which require one to recall directions at each intersection in the same order as during encoding in the route-learning task and allow one to recall words in any order in the list-learning tasks, respectively. Further studies are needed to investigate whether these methodological differences can account for differences in serial position effects in cognitively impaired older adults in word-list and route-learning studies.

In the route-retracing task, where participants navigated from the end of the route back to the start location, we observed navigation deficits in aMCI patients that were even more pronounced in patients with mild AD dementia. This finding is consistent with a previous study showing navigation deficits in patients with MCI and mild AD, which were more frequent in the latter group, when navigating a route in the reverse direction in a real-space hospital lobby [50]. CN older adults gradually improved their performance across three experimental sessions, while patients with aMCI showed slower learning as they only improved between the first and last sessions. Patients with mild AD dementia did not show any learning and performed at chance level in all sessions. Successful navigation in this task requires creating a mental representation of the environment or the ability to imagine the environment from different viewpoints as egocentric strategies do not directly support route retracing [38]. About 94% of CN older adults, 86% of aMCI patients, and 59% of patients with mild AD dementia reported that they remembered a sequence of turns (i.e., the sequence-of-directions strategy) or created associations between landmarks and movement directions (i.e., the stimulus–response strategy) in the encoding phase and mirrored the direction of the turn required at each intersection when retracing a route, whereas none of the participants reported creating a cognitive map to solve the task. Worse performance in participants with cognitive deficits in this retracing task may thus be caused by the inaccurate alignment of the viewpoint in the test phase with the encoded viewpoint, i.e., by perspective shift deficits that have been reported in patients with aMCI and mild AD, with more pronounced impairment in the latter group [28]. 

It is noteworthy that 41% of patients with mild AD dementia did not report any specific strategy (i.e., used the non-specific strategy). The use of this non-specific strategy was associated with worse task performance compared to adopting the sequence-of-directions strategy. Therefore, the inability to adopt an effective non-egocentric strategy to solve the task could contribute to severe route-retracing deficits and the absence of learning in patients with mild AD dementia, and is in line with previous findings of a strong preference for egocentric (i.e., extrahippocampal) strategies in AD patients [26].

We also observed serial position effects in this task. Specifically, we found a recency effect in all groups, where participants showed the best performance in the last encoded (i.e., first recalled) intersection. In addition, we observed a primacy effect in CN older adults with better performance in the first encoded (i.e., last recalled) intersection. To the best of our knowledge, the serial position effect has not been studied in route-retracing tasks. A recent study using a different paradigm reported primacy and recency effects for landmarks in a route-learning task in CN older adults [41]. Our findings of prominent recency and reduced primacy effects in cognitively impaired older adults are in line with studies showing reduced primacy effect in verbal memory tests in patients with MCI and AD dementia [58] and in non-verbal memory tests in the latter group [59].

In the directional-approach task, the participants were required to encode spatial relationships of landmarks (houses) at an intersection in relation to the direction from which the intersection was approached originally. Here we observed navigation deficits in patients with aMCI and mild AD dementia compared to CN older adults. This is in line with a previous study showing navigation deficits in patients with MCI and mild AD that were more pronounced when approaching an intersection from a different direction than during learning [50]. In all groups, performance decreased when the approach direction in the test phase was misaligned with the encoding phase by 180° compared to 90°. This corroborates similar findings of a previous study with the same paradigm in CN older adults [38] and supports earlier notions that perspective-taking abilities are required to solve this task [48]. It should be noted that patients with aMCI and mild AD dementia performed at and below the chance level, respectively, when the direction in the test phase was misaligned by 180°. Our findings thus indicate that patients with aMCI and mild AD dementia show perspective-taking deficits similar to what was previously reported in a virtual arena task [28]. 

The CN normal older adults most frequently reported that they remembered the positions of unique houses at the intersection (i.e., the unique-houses strategy), while aMCI patients reported with a similar frequency that they used unique houses and non-unique houses or more than two houses (i.e., unique-houses and more-houses strategies). Almost half of the patients with mild AD dementia did not report any specific strategy (i.e., used the non-specific strategy). It is important to note that the use of the unique-houses strategy compared to the adoption of any other strategy was associated with better task performance. Using an effective strategy was associated with better performance even in the mild AD dementia group. These findings indicate that successful navigation in this task requires an engagement of allocentric processes including knowledge about the spatial relationships between landmarks (i.e., unique houses) and a place (i.e., street from which the intersection was approached) that were reported to be impaired in patients with aMCI and mild AD dementia [26].

Further, we assessed differences in spatial navigation performance in women and men showing that gender did not influence performance in route-repetition and route-retracing tasks. However, the effect of gender was found in the directional-approach task where women had worse performance than men across all diagnostic groups. This is consistent with our previous findings where the perspective-taking deficit was more pronounced in women than men [28].

Finally, we explored the associations of spatial navigation performance between the three navigation tasks and with cognitive functions. We found correlations between the tasks in aMCI patients but not in CN older adults and patients with mild AD dementia. This finding may reflect greater variability of performance in aMCI patients and supports the previously described heterogeneity of MCI patients who may greatly vary with respect to spatial navigation performance [18,60] and the number, type, and severity of impairment of individual cognitive functions [47,61]. Spatial navigation performance correlated with memory, executive, and language functions in CN older adults, memory in aMCI patients, and executive function in patients with mild AD dementia. These findings indicate that specific cognitive resources including the use of effective memory strategies [62] and executive skills (e.g., planning and strategy selection) [63] may contribute to successful spatial navigation. They are also congruent with previous research associating more effective spatial navigation in various tasks with better memory, executive, and language functioning in CN older adults [63,64,65,66,67] and better memory and executive functioning in patients with MCI and AD dementia [51,68,69]. 

The strengths of the current study are the fact that we explored three different spatial navigation tasks that rely on different cognitive processes in the same participants. Specifically, we recruited a sufficient number (*n* = 219) of clinically well-defined CN older adults and patients with cognitive impairment including patients with aMCI and mild AD dementia. We also used an established, realistic-looking, and ecologically valid method for spatial navigation testing, the Navigation Test Suite, along with detailed cognitive assessment. However, this study is not without its limitations. First, we have not examined structural and functional alterations in specific brain regions that support spatial navigation to reveal the underlying mechanisms of the observed behavioral findings, which should be a focus of future studies. Second, we have not analyzed specific biomarkers including cerebrospinal fluid analysis and amyloid or tau positron emission tomography imaging to confirm the clinical diagnosis of AD in patients with mild dementia and aMCI. Third, in each task, we used separate ANCOVA analyses for each hypothesis, although using one ANCOVA would be a more appropriate statistical method. However, such analysis would make the results difficult to interpret and, importantly, it would significantly reduce the statistical power in our sample size. Fourth, the cross-sectional design did not allow for evaluating the changes in spatial navigation performance over time, but longitudinal follow-up is ongoing.

## 5. Conclusions

In conclusion, the current study demonstrated spatial navigation deficits in route learning, route retracing, and wayfinding/perspective-taking in patients with aMCI that are even more pronounced in patients with mild AD dementia. CN older adults were able to learn in both route-learning and route-retracing tasks, while patients with aMCI and mild AD dementia were able to gradually improve their performance only in a route-learning task when the number of intersections did not exceed the spatial span of AD patients. Patients with aMCI showed slower learning in a route-retracing task, while patients with mild AD dementia did not learn and performed at chance level throughout the experiment. Greater misalignment in a wayfinding/perspective-taking task was associated with worse performance in both typical and atypical aging. CN older adults and aMCI patients were able to adopt specific strategies in route-learning and route-retracing tasks, while patients with mild AD dementia more frequently used non-specific strategies. Patients with aMCI used specific landmarks for navigation in a wayfinding/perspective taking task less frequently than CN older adults. This was even more pronounced in patients with mild AD dementia. Adopting specific strategies and using specific landmarks is crucial for successful navigation and was associated with better spatial navigation performance in typical and atypical aging but this may not apply to route-learning performance in patients with mild AD dementia. Serial position effects varied with respect to cognitive performance and type of the task. Specifically, in the route-repetition task, we found a primacy effect in both typical and atypical aging, while primacy and recency effects were present in the route-retracing task in CN older adults together with a recency effect in patients with aMCI and mild AD dementia. More effective spatial navigation performance was associated predominantly with better memory and executive functions. These findings indicate different aspects of spatial navigation and visuospatial strategies in typical and atypical aging that can be revealed by a realistic-looking and ecologically valid spatial navigation test suite.

## Figures and Tables

**Table 1:** Characteristics of study participants.

Variables	CN (*n* = 78)	aMCI (*n* = 75)	Mild AD Dementia (*n* = 66)	*p* Values	Effect Sizes
Age (years)	68.22 (6.80)	71.49 (7.38) *	75.91 (8.06) ***^++^	<0.001	0.15
Women, n (%)	56 (72)	33 (44)	42 (64)	0.002	0.24
Education (years)	15.97 (2.03)	14.91 (2.52) *	14.29 (3.01) ***	<0.001	0.07
MMSE (score)	29.06 (1.25)	27.53 (1.91) ***	22.55 (2.75) ***^+++^	<0.001	0.65
GDS-15 (score)	2.04 (2.25)	2.49 (2.29)	2.90 (2.61)	0.130	0.02
BAI (score)	7.46 (6.58)	7.41 (6.81)	7.56 (6.44)	0.993	0.00
RAVLT 1-5 (score)	55.83 (6.86)	35.19 (8.49) ***	27.08 (6.89) ***^+++^	<0.001	0.71
RAVLT 30 (score)	11.97 (1.91)	4.29 (2.92) ***	2.29 (3.75) ***^++^	<0.001	0.71
LM-IR (score)	17.42 (3.64)	11.91 (4.37) ***	7.16 (3.40) ***^+++^	<0.001	0.53
LM-DR (score)	16.19 (3.87)	8.35 (5.75) ***	2.84 (4.50) ***^+++^	<0.001	0.56
Stroop (seconds)	28.34 (8.10)	38.69 (15.78) **	59.87 (29.82) ***^+++^	<0.001	0.31
TMT A (seconds)	37.97 (10.89)	48.47 (18.33) *	72.60 (41.00) ***^+++^	<0.001	0.24
TMT B (seconds)	86.19 (31.46)	140.04 (70.87) ***	243.44 (72.28) ***^+++^	<0.001	0.52
COWAT (score)	51.21 (10.20)	40.38 (10.51) ***	33.06 (14.15) ***^++^	<0.001	0.29
ROCFT-C (score)	31.18 (3.30)	26.69 (4.94) ***	25.19 (7.36) ***	<0.001	0.20
ROCFT-R (score)	19.42 (5.61)	9.20 (5.69) ***	4.66 (5.20) ***	<0.001	0.55
DSF (score)	9.65 (2.06)	8.83 (1.79) *	8.04 (1.88) ***	<0.001	0.10
DSB (score)	6.88 (1.75)	5.84 (1.76) ***	4.88 (1.34) ***^++^	<0.001	0.19
CDT (score)	15.19 (1.13)	14.28 (1.78) **	12.82 (2.32) ***^+++^	<0.001	0.22
SVF Animals (score)	27.32 (4.84)	19.72 (5.41) ***	14.30 (5.47) ***^+++^	<0.001	0.50
BNT (no. of errors)	1.53 (1.93)	4.01 (3.13) ***	7.26 (3.72) ***^+++^	<0.001	0.37

Demographic and cognitive characteristics. Values are mean (SD) except for gender. *p* Values refer to the main effect across all groups. For *p* values indicating the level of significance compared to the CN group: * *p* < 0.05; ** *p* < 0.01; *** *p* < 0.001 and compared to the aMCI group: ^++^ *p* < 0.01; ^+++^ *p* < 0.001. Effect sizes were calculated as Cramér’s V for the χ^2^ test (gender) and partial eta-squared for one-way and mixed analyses of variance (all other variables). CN, cognitively normal; aMCI, amnestic mild cognitive impairment; mild AD dementia, mild Alzheimer’s disease dementia; MMSE, Mini-Mental State Examination; GDS-15, Geriatric Depression Scale 15-item version; BAI, Beck Anxiety Inventory; RAVLT, Rey Auditory Verbal Learning Test; RAVLT 1-5, trials 1 to 5 total; RAVLT 30, delayed word recall after 30 min; LM-IR, Logical Memory—Immediate Recall; LM-DR, Logical Memory—Delayed Recall; Stroop, Prague Stroop Test—colors; TMT A and B, Trail Making Tests A and B; COWAT, Controlled Oral Word Association Test (Czech version with letters N, K and P); ROCFT-C, Rey–Osterrieth Complex Figure Test—the Copy condition; ROCFT-R, Rey–Osterrieth Complex Figure Test—the Recall condition after 3 min; DSF, Digit Span Forward total score; DSB, Digit Span Backward total score; CDT, Clock Drawing Test—Cohen’s scoring; SVF, Semantic Verbal Fluency; BNT, Boston Naming Test.

## Data Availability

The data presented in this study are available on request from the corresponding author. The data are not publicly available due to privacy restrictions.
